# Limited impact of vector control on the population genetic structure of *Glossina fuscipes fuscipes* from the sleeping sickness focus of Maro, Chad[Fn FN1]

**DOI:** 10.1051/parasite/2024013

**Published:** 2024-03-06

**Authors:** Sophie Ravel, Adeline Ségard, Brahim Guihini Mollo, Mahamat Hissène Mahamat, Rafael Argiles-Herrero, Jérémy Bouyer, Jean-Baptiste Rayaisse, Philippe Solano, Mallaye Pèka, Justin Darnas, Adrien Marie Gaston Belem, Wilfrid Yoni, Camille Noûs, Thierry de Meeûs

**Affiliations:** 1 Intertryp, Université de Montpellier, Cirad, IRD Montpellier France; 2 Institut de Recherche en Élevage pour le Développement (IRED) Ndjaména Chad; 3 Insect Pest Control Laboratory, Joint Food and Agriculture Organization of the United Nations/International Atomic Energy Agency Program of Nuclear Techniques in Food and Agriculture A-1400 Vienna Austria; 4 UMR Astre, Cirad, Plateforme Cyroi 2 rue Maxime Rivière 97491 Sainte-Clotilde La Réunion France; 5 Centre International de Recherche Développement sur l’Élevage en zone Subhumide (Cirdes) Bobo-Dioulasso Burkina Faso; 6 Programme National de Lutte contre la THA (PNLTHA) Ndjaména Chad; 7 Université Nazi Boni Bobo-Dioulasso Burkina Faso; 8 Cogitamus Laboratory France, https://www.cogitamus.fr/

**Keywords:** Tsetse flies, Dispersal, Trypanosomosis, Control

## Abstract

Tsetse flies (genus *Glossina*) transmit deadly trypanosomes to human populations and domestic animals in sub-Saharan Africa. Some foci of Human African Trypanosomiasis due to *Trypanosoma brucei gambiense* (g-HAT) persist in southern Chad, where a program of tsetse control was implemented against the local vector *Glossina fuscipes fuscipes* in 2018 in Maro. We analyzed the population genetics of *G. f. fuscipes* from the Maro focus before control (T0), one year (T1), and 18 months (T2) after the beginning of control efforts. Most flies captured displayed a local genetic profile (local survivors), but a few flies displayed outlier genotypes. Moreover, disturbance of isolation by distance signature (increase of genetic distance with geographic distance) and effective population size estimates, absence of any genetic signature of a bottleneck, and an increase of genetic diversity between T0 and T2 strongly suggest gene flows from various origins, and a limited impact of the vector control efforts on this tsetse population. Continuous control and surveillance of g-HAT transmission is thus recommended in Maro. Particular attention will need to be paid to the border with the Central African Republic, a country where the entomological and epidemiological status of g-HAT is unknown.

## Abstract (fr)

Les mouches tsé-tsé (genre *Glossina*) transmettent des trypanosomes mortels aux populations humaines ainsi qu’aux animaux domestiques en Afrique sub-saharienne. Certains foyers de la trypanosomiase humaine Africaine due à *Trypanosoma brucei gambiense* (THA-g) persistent au sud du Tchad, où un programme de lutte antivectorielle a été mis en place contre le vecteur local de la maladie, *Glossina fuscipes fuscipes*, en particulier à Maro en 2018. Nous avons analysé la structure génétique des populations de *G. f. fuscipes* de ce foyer à T0 (avant lutte), une année après le début de la lutte (T1), et 18 mois après (T2). La plupart des mouches capturées après le début de la lutte ont montré un profil génétique local (survivants locaux), mais quelques-unes d’entre elles présentaient des génotypes d’individus atypiques. Par ailleurs, la présence de perturbations des signatures d’isolement par la distance (augmentation de la distance génétique avec la distance géographique), l’absence de signature génétique d’un goulot d’étranglement, et un accroissement de la diversité génétique entre T0 et T2 sont des arguments forts en faveur de la recolonisation de la zone par des mouches d’origines variées, tout en témoignant des effets limités de la campagne de lutte dans ce foyer. Ces résultats conduisent à recommander une lutte et une surveillance continues dans le foyer de Maro. Une attention particulière devra par ailleurs être prêtée à l’autre côté de la rive, située côté République Centre Africaine, dont le statut épidémiologique reste inconnu concernant les tsé-tsé et la THA-g.

## Introduction

Tsetse flies (genus *Glossina*) transmit deadly trypanosomes to human populations and domestic animals in sub-Saharan Africa, causing Human African Trypanosomiasis due to *Trypanosoma brucei gambiense* Dutton, 1902 (g-HAT) or sleeping sickness, and Animal African Trypanosomosis (AAT) or nagana. There is no vaccine, and treatment remains difficult in humans despite recent progress [24]. The WHO aims at interrupting transmission of g-HAT due to *T. b. gambiense* by 2030 [27, 34]. Despite intensive control programs, some g-HAT foci persist in different zones of Sub-Saharan Africa. In the south of Chad, tsetse control has been implemented since 2014 in the Mandoul focus [38] and since 2018 in Maro [42] against *Glossina fuscipes fuscipes* Newstead 1910, in addition to diagnostic and treatment activities. Insecticide-impregnated tiny targets have been used, with a subsequent substantial decrease of human infections attributable at 63% to tsetse control in the focus of Mandoul [38]. Nevertheless, to understand and predict the sustainability of such disease control programs, it is necessary to expand the body of knowledge on the population biology of the vector, in particular subpopulation sizes, dispersal capacities, and genetic relatedness between flies captured before and after control, to assess resurgence risks. This can be relatively easily addressed with population genetics tools and polymorphic genetic markers such as microsatellites [18]. Such information can be used to inform the tsetse control strategy, *i.e.*, local eradication can be considered only if the tsetse target population is isolated [4, 57], whereas alternative solutions should be used otherwise.

**Figure 1:**
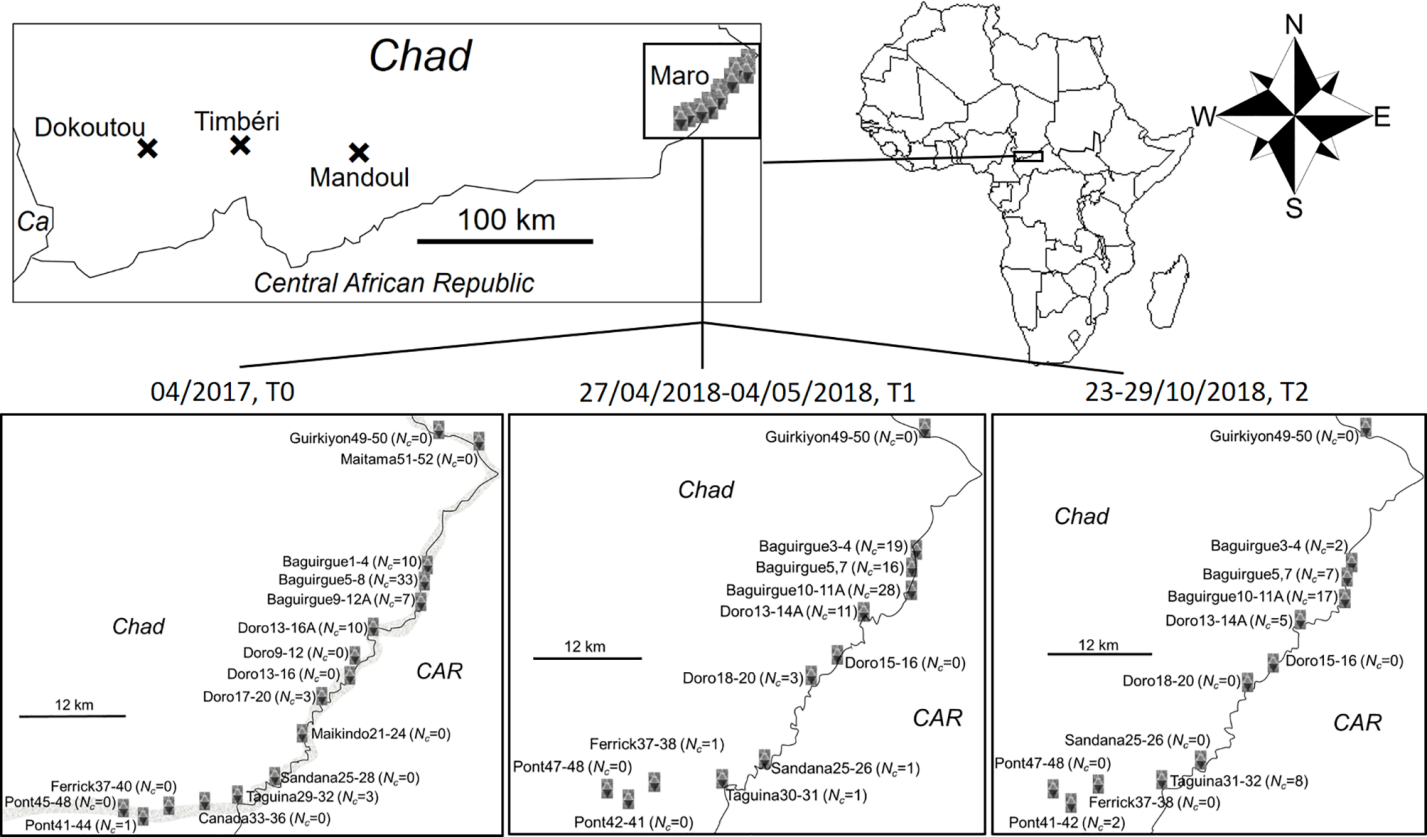
Location of sampling sites and traps for *Glossina fuscipes fuscipes* in southern Chad [46] and specifically in Maro before (T0) and during control (T1 and T2). Numbers of flies trapped are indicated (see also Table 1) (*Ca*: Cameroon; *CAR*: Central African Republic). Traps too close to each other (less than 400 m apart) were combined, *e.g*., Doro 13–16 contained traps 13, 14, 15 and 16 in Doro.

In a previous study [50], we found that *G. f. fuscipes* in the different zones investigated in southern Chad (see Figure 1 in cited reference) were genetically quite isolated. We also found large within-zones dispersal distances (up to 30 km/generation, depending on the zone), and rare exchange between zones was suggested, probably via the gallery forests at the southernmost part of the investigated areas that were poorly investigated if not unknown. This was particularly true for the borders with neighboring countries like the Central African Republic (CAR), a country where the security and humanitarian situation have recently significantly worsened. The g-HAT focus of Maro, which is close to this border, indeed presented the highest genetic heterogeneity, compatible with recurrent immigrations from more or less remote sites that did not belong to the spectrum of genetic variation observed in the different zones explored so far [50]. In the present study, we specifically analyzed the population genetics of *G. f. fuscipes* of Maro, one of the main g-HAT foci of the country, before tsetse control [50] and after control had begun (present study). This study, among others, aimed at measuring the impact of vector control on population genetics parameters in the particular context of HAT foci of southern Chad. A study of this type was possible only in Maro, as tsetse can no longer be captured in the other focus Mandoul [38]. We used nine microsatellite loci on a total sample of 169 tsetse flies and a population genetics data analysis to check what kind of flies are caught in the traps after the beginning of the control campaign, and estimated its effect on effective population density, dispersal distances, and bottleneck signatures. We then discuss the consequences of the results observed in terms of tsetse control strategies in this geographic area.

## Material and methods

### Ethical statement

A prior informed consent (PIC) was obtained from the local focal point and a mutually agreed terms (MAT) form was written and approved between Chadian laboratories and French laboratories involved in the study for the use of the genetic diversity found in tsetse flies from Chad.

### Origin of the samples

Tsetse flies were captured in biconical traps [8] deployed for 48 h. Trapped flies were processed as described elsewhere [49] and their legs stored in 95% alcohol. Details of traps deployed in Maro at different sites and dates, and numbers of captured flies are presented in Figure 1. Detailed data with genotypes of individuals are available in the Supplementary File S1. T1 is 12 months after T0, and T2 is 18 months after T0, all during the dry season (see Figure 1). With a two-month generation time, as assumed previously [50], these dates would correspond to generation or cohort C0, C6 and C9. Vector control was undertaken with deltamethrin-impregnated tiny targets [38, 41, 42, 52]. It began in February 2018, with 2,031 tiny targets deployed along the Grande Silo and Chari Rivers across around 100 km [42] (*i.e.*, around one trap every 50 m on average, depending on accessibility and vegetation cover).

The number of sampled flies (females, males and total), the corresponding time after control, the number of genotyped flies, the densities of captured flies, and the observed sex-ratio are presented in Table 1. For T1 and T2, captured flies corresponded to the totality of flies trapped in all the 60 sentinel traps deployed along the Grande Sido and Chari rivers.

**Table 1:** Time before (T0) or during control (T1 and T2), number of females (*N*_f_), males (*N*_m_), total number (*N*_t_) of *fuscipes fuscipes* trapped in the Maro focus, southern Chad, and number of genotyped individuals (*N*_g_). The sex-ratio (SR = *N*_m_/*N*_f_), with exact *p*-values for significant deviation from even SR (two-sided exact binomial test), densities of captured flies (*D*_*c*_X_) (individuals per km^2^, X stands for GPS or GEPL), computed as *N*_t_/*S*, and the trap-based effective population sizes and densities (*D*_e_traps-X_ = *N*_e_traps_/*S*_X_) and its range (bracketed) are also given (see below for *N*_e_ computations).

	T0	T1	T2
*N*_f_	49	54	18
*N*_m_	18	26	23
*N*_t_	67	80	41
*N*_g_	63	66	40
SR	0.3673	0.4815	1.2778
*p*-value	0.0002	0.0023	0.5327
*N*_e_traps_ [range]	28 [17, 46]	187 [14, 523]	18 [15, 23]
*S*_GPS_ (km^2^)	128		
*D*_*c*_GPS_ (/km^2^)	0.52	0.63	0.32
*S*_GEPL_ (km^2^)	308		
*D*_*c*_GEPL_ (/km^2^)	0.217	0.26	0.13
*D*_e_traps-GPS_ (/km^2^) [range]	0.22 [0.13, 0.36]	1.46 [0.11, 4.09]	0.14 [0.12, 0.18]
*D*_e_traps-GEPL_ (/km^2^)	0.09 [0.05, 0.15]	0.61 [0.05, 1.70]	0.06 [0.05, 0.07]

Deviation of the sex-ratio from 1 (even sex-ratio) was tested with a two-sided exact binomial test with R [53] (command binom.test). Significance of variations of the sex-ratio from one time to another was tested with Fisher’s exact tests under the R-commander (rcmdr) package [25, 26] for R. In case of paired comparisons (between times after control), we adjusted *p*-values with the Benjamini and Yekutieli procedure [2] with R (command p.adjust). Densities of trapped flies (*D*_t_) were computed as the total number of flies captured (*N*_t_, as defined in Table 1), divided by the surface area of the zone populated by tsetse flies in this HAT focus. This surface area was first computed with Karney’s algorithm [37] with the package geosphere (command areaPolygon) [33] for R (see Appendix A). We used the polygon defined by the GPS coordinates (in decimal degrees) of all traps found with at least one fly during all the sampling campaigns. These traps were ordered in the dataset following a southwest-east-west transect to obtain a reasonably regular polygon (*S*_GPS_ = 128.3 km^2^). We also used the “Polygon” function of GoogleEarth Pro to determine the surface area of the polygon containing all favorable sites surrounding the river bordering Chad and the CAR in that zone, the Grande Silo river (*S*_GEPL_ = 279 km^2^). Comparisons for number of flies captured between different times after the beginning of control were undertaken with a one-sided (captured flies should decrease) Wilcoxon signed rank test for paired data with rcmdr, the paring unit being the site as defined in Figure 1 (see also Supplementary File S1).

### Microsatellite markers

We used a total of nine di-nucleotidic microsatellite loci (GFF3, GFF4, GFF8, GFF12, GFF16, GFF18, GFF21, GFF23, and GFF27) with primers designed from a previously built microsatellite bank of *G. f. fuscipes* [51]. All markers were autosomal (*i.e.*, not on the X chromosome).

### Genotyping

Legs from captured flies were received at the Montpellier laboratory. Three legs from each *G*. *f. fuscipes* individual were subjected to chelex treatment as previously described [49] in order to obtain DNA for further microsatellite genotyping.

After PCR amplification of microsatellite loci, allele bands were routinely resolved on an ABI 3500XL sequencer. This method enables multiplexing by the use of four different dyes. Allele calling was done using GeneMapper 4.1 software and the size standard GS600LIZ short run. A total of 169 individuals were genotyped (Table 1).

### Structure of the data

Data were sorted according to the trapping time (T0, T1 and T2), then site (six sites: Baguirgue, Doro, Ferrick, Pont, Sandana, and Taguina) (Figure 1), then according to the sub-site as defined in Figure 1 (traps that were less than 400 m apart belonged to the same sub-site) and the individual trap (see Figure 1 and Supplementary File S1). Following this, in subsequent analyses, a subsample was defined according to the time (T0, T1 and T2), and to one of these pre-defined geographic subdivisions (site, sub-site and trap), and thus assumed as a subpopulation. Raw data are available in Supplementary File S1.

All genetic data were typed in the Create [10] format and converted by this software into the needed formats.

Before control, only the trap appeared as a significant (though feeble) level of subdivision in Maro, while we also found some evidence of (almost) free dispersal across the whole focus (~30 km long) [50]. We considered only individual traps or the whole focus as the subpopulation units in further analyses.

### Testing the quality of genetic markers

We first studied the statistical independence of loci with the *G*-based test for linkage disequilibrium (LD) across individual traps implemented in Fstat 2.9.4 [29], updated from [30], with 10,000 randomizations. This procedure is indeed the most powerful way to combine tests across subsamples [16]. There were as many non-independent tests as there were locus pairs (here 36 pairs). The 36 test series were adjusted with the Benjamini and Yekutieli (BY) false discovery rate (FDR) procedure for non-independent test series [2] with R.

Deviation from local panmixia, absence of subdivision, and deviation from global panmixia were measured by Wright’s *F*_IS_, *F*_ST_ and *F*_IT_, respectively [69]. These were estimated with Weir and Cockerham’s unbiased estimators [68] and their significance tested with 10,000 randomizations of alleles between individuals within subsamples (for panmixia), of individuals between subsamples (for subdivision), and of alleles between individuals within the total sample, respectively. These tests were undertaken with *F*_stat_. The statistics used were the *F*_IS_ estimator, *G* [31] and *F*_IT_ estimator, respectively. Default testing is unilateral (heterozygote deficit) for *F*_IS_ and *F*_IT_. The bilateral *p*-value was obtained by doubling the *p*-value if it was below 0.5, or doubling 1-*p*-value if above 0.5. When needed, we compared *F*_IS_ and *F*_IT_ with a one-sided (*F*_IS_ < *F*_IT_) Wilcoxon signed rank test for paired data with rcmdr. In that case, the pairing unit was the locus.

In case of significant heterozygote deficit, we looked for short allele dominance (SAD), stuttering, null alleles, and Wahlund effects, as described in previous studies [13, 15, 19, 39]. A Wahlund effect occurs when a subsample contains individuals from different groups that do not share the same allelic frequencies. This phenomenon displays several population genetics signatures. The simplest is an increase of *F*_IS_, but other signatures may depend on what parameter is looked for and where (LD, effective population size, subdivision estimate, *etc*.). For *F*_IS_ / missing data and *F*_IT_ or *F*_IS_/allele size correlation tests, to test for null allele or SAD signatures, respectively we used one-sided Spearman’s rank correlation tests with rcmdr, with a positive and a negative expected correlation, respectively. In case of doubt, we also undertook the regression *F*_IS_ ~ size of allele *i*, weighted with the product *p*_*i*_(1 − *p*_*i*_) [17]. Null allele frequency estimations were assessed with the EM algorithm [22] with FreeNA [9], except for T0 that was analyzed with Brookfield’s second method [5] under MicroChecker [59], and published elsewhere [50]. The goodness of fit of expected null homozygotes and observed missing data was tested with a one-sided (there are not enough observed missing data) exact binomial test with R. Stuttering signatures were detected and corrected with the spreadsheet method [15, 19], except for T0 that was analyzed and published elsewhere [50].

Jackknife over subsamples provided a standard error for *F*-statistics: StdrdErrFIS, StdrdErrFST and StdrdErrFIT. This enabled computing 95% confidence intervals (95%CI) of *F*-statistics as described in [18] to measure locus variation across subsamples. As it uses the student *t* distribution (assuming normality, which is obviously not the case here), these 95%CI had only an illustrative purpose. The 95%CIs of *F*-statistics were also obtained with 5,000 bootstraps over loci, as described in [18, 14]. This procedure assumes no particular distribution and thus has statistical utility.

LD tests, *F*-statistic estimates and testing, jackknives and bootstraps were undertaken with Fstat 2.9.4 [29] updated from [30].

### Global level of subdivision

Because of the presence of null alleles, *F*_ST_ was estimated with the ENA correction with FreeNA [9], for which we recoded missing data as homozygous for null alleles (coded 999, as recommended). We labelled this new estimate as *F*_ST_FreeNA_. In microsatellite loci, because of high mutation rates and excesses of polymorphism that results from it, the maximum possible value is lower than unity for *F*_ST_ (*F*_ST_max_ < 1) [32]. To correct this bias, we can either divide the actual estimator by the maximum possible value given the polymorphism observed within subsamples, or use *G*_ST_″ = [*n*(*H*_T_ − *H*_S_]/[(*nH*_T_ − *H*_S_)(1 − *H*_S_)] [37], where *H*_S_ and *H*_T_ are Nei’s [43] unbiased estimators of genetic diversity within subpopulations individual (traps) and in the total population (Maro focus), respectively and *n* are the number of subsamples (traps) used to compute these quantities. Wang’s criterion [63] can be used to determine which of the two approaches is more appropriate. If the correlation between Nei’s *G*_ST_ and *H*_S_ is strongly negative, then *F*_ST_ based standardizations are more accurate, otherwise *G*_ST_″ should be used. We computed the standardized estimator of *F*_ST_ using Recodedata [40] to compute a maximum possible *F*_ST_FreeNA_max_. We then obtained the standardized *F*_ST_FreeNA_′ = *F*_ST_FreeNA_/*F*_ST_FreeNA_max_. In this case, we obtained 95%CI with 5,000 bootstraps over loci. These standardized subdivision measures could then be used to compute the effective number of immigrants within subpopulations as *N*_e_*m* = (1-*F*_ST_′)/(4*F*_ST_′), where *F*_ST_′ stands for *G*_ST_″ or *F*_ST_FreeNA_′ (depending on Wang’s criterion), and assuming an Island model of migration. Since *G*_ST_” cannot be corrected for null alleles and does not allow 95%CI computations, we computed *F*_ST_FreeNA_′ even in situations in favor of *G*_ST_”.

### Effective population sizes

In a previous study, we found that the only relevant hierarchical level of population subdivision, within the focus as a whole, was the trap (*i.e.*, no significant effect of other levels in the hierarchy: subsites and sites) [50]. Effective population sizes were estimated in each trap with five different methods. The first method was the linkage disequilibrium (LD) method [66] adjusted for missing data [46], and the second was the coancestry method [45]. These two methods were both implemented with NeEstimator version 2.1 [23]. The third was the within and between loci correlations method [61] computed with Estim 1.2 [60] updated from [62]. The fourth was the heterozygote excess method from De Meeûs and Noûs, using values obtained for each locus in each trap, and averaging the results over loci [20]. The last method was the sibship frequency method [64] with Colony [35]. For the LD method, we retained only data with minimum allele frequency 0.05 as recommended in the NeEstimator manual. For each method, we averaged *N*_e_ across traps (excluding “infinite” results). We also retained minimum and maximum values across the four methods used. We finally computed the grand average and average minimum and maximum *N*_e_ across methods, weighted by the number of usable values. Taking into account the lack of subdivision at T0 [50], we considered that these trap-based averages (*N*_e_traps_) corresponded to the effective trap-based population size of the focus as a whole.

Due to the small subsample sizes when considering traps as subpopulation units, trap-based estimates displayed highly variable and often not computable results at T0 [50]. Since subdivision was weak in Maro [50], the whole focus could be approximated as a single subpopulation. We used this property to compute effective population sizes at this scale, *N*_e_all_, for T0, T1 and T2, corresponding to generations 0, 6 and 9, respectively with their 95%CIs for LD, coancestries and sibship methods (parametric for LD, jackknife for Coancestries), and minimax for the *F*_IS_ based method (bootstrap values not usable). We also used temporal methods: Maximum likelihood method (ML) averaged across the three samples and 95%CI with MNE [65]; Pollak’s method (PM), Nei and Tajima’s method (NT), and Jorde and Ryman’s method (JR) [36, 44, 48] implemented in NeEstimator between each pair of samples (T0/T1, T0/T2, and T1/T2) with parametric 95%CI. For each subsample pair, we computed the average averaged *N*_e_ obtained across methods. For 95%CI, infinite upper limits were replaced by repeating the value obtained for *N*_e_. This was made to prevent averages to outreach the average upper limit.

### Effective population densities

We computed the surface area of the population (*S*) with the command “areaPolygon” of the package geosphere of R with the GPS coordinates in decimal degrees of all traps with a genotyped fly over all seasons of trapping (T0, T1 and T2). We labelled this surface area *S*_GPS_ = 128 km^2^. Since the actual tsetse population obviously extends beyond the border with the CAR, we also drew a polygon that approximately contained all the forest gallery on both sides of the border with Google Earth Pro: *S*_GEPL_ = 279 km^2^. The effective population density was then estimated as *D*_e_traps-X_ = *N*_e_traps_/*S*_X_ where X stands for GPS or GEPL.

### Isolation by distance

Isolation by distance was tested inside each cohort (T0, T1 and T2) separately. It was measured and tested with Rousset’s model of regression in two dimensions *F*_ST_R_ = *a* + *b* × ln(*D*_Geo_) [54]. In this equation, *F*_ST_R_ = *F*_ST_/(1 −*F*_ST_) is Rousset’s genetic distance between two subsamples (traps), *a* and *b* are the intercept and the slope of the regression, respectively and ln(*D*_Geo_) is the natural logarithm of the geographic distance between two traps. Geographic distances were computed with the distGeo command of the geosphere package in R. The significance of the regression was tested by 5,000 bootstraps over loci that provided a 95%CI of the slope. Because null alleles were present, we recoded all blank genotypes as homozygous profiles for allele 999 and used the ENA correction as recommended [9] to compute *F*_ST-FreeNA_. This was undertaken with FreeNA [9] and 5,000 bootstraps over loci. In case of significance, *i.e.*, if the 95%CI of the slope of Rousset’s regression is above 0, the neighborhood size and number of immigrants coming from direct neighbors and entering a subpopulation at each generation [54] was computed as (in two dimensions) *Nb* = 4*πD*_e_$\bar{\sigma^{2}}$=1/*b*, and *N*_e_*m* = 1/(2*πb*), respectively [54, 67]. In these formulae, *D*_e_ is the effective population density, $\bar{\sigma^{2}}$ is the average of squared axial distances between adults and their parents, and *b* is the slope of Rousset’s regression model [54] for isolation by distance.

In case of non-significance, we also undertook a Mantel test using the Cavalli-Sforza and Edwards’ chord distance *D*_CSE-FreeNA_ [7], computed with the INA correction for null alleles [9] with FreeNA and 10,000 randomizations with the “Mantelize it” menu of Fstat. This genetic distance can indeed prove more powerful in case of weak signals [55]. Mantel test in Fstat is two sided. Since we expected a positive correlation, we computed the one-sided *p*-value as half the *p*-value obtained for a positive correlation or 1 − *p*-value/2 otherwise.

### Dispersal distances

The average distance between adults and their parents was extracted with the equation (*e.g*., [21]):

$$\delta \approx 2\sqrt{\frac{1}{4\pi bD_{e}}}.$$

In this equation, *b* is the slope of Rousset’s regression for isolation by distance, and *D*_e_ is the average effective population density. This quantity is only accurate when dispersal distances follow a symmetrical distribution with a strong kurtosis. In any other case, like skewed distributions (right or left), or platykurtic distributions, *δ* will be slightly overestimated. Since there is also a lack of accuracy for *D*_e_, *δ* corresponded more to an order of magnitude than a precise estimate of dispersal distances.

### Genetic differentiation between trapping times

Genetic differentiation between trapping times (T0, T1 and T2) was tested with the *G*-based test between paired dates of the same trap when available and with 10,000 permutations of individuals between the two dates with the pairwise test of differentiation of Fstat. To get a global *p*-value across traps within each comparison type (*i.e.*, T0/T1, T0/T2 and T1/T2) we used the generalized binomial procedure [58] with MultiTest V1.2 [16]. The three *p*-values obtained were then submitted to the BY correction with R to take into account the FDR in test series with dependency. Paired *F*_ST_ were estimated between relevant pairs of time for the same trap, with the INA correction for null alleles and 5,000 bootstraps over loci to get 95%CI. We averaged these values over traps for each pair type.

We also undertook these differentiations measures and tests assuming that Maro is a single population of *G. f. fuscipes*, as suggested in [50]. We undertook these calculations between each temporal sample T0, T1 and T2.

### Factorial correspondence analysis (FCA)

In order to visualize how the genetic information of the different individuals distribute relative to each other’s and particularly their position after vector control had begun (T1-2) as compared to T0 samples, we undertook two types of analyses: factorial correspondence analysis for genotypic data (FCA) [56], where the values of inertia along each principal axis can be seen as *F*_ST_ combinations of different alleles of the different loci. This analysis was undertaken with Genetix [1]; significance of the first axes tested with the broken stick criterion [28].

### Bottleneck detection

We used the algorithm developed by Cornuet and Luikart [11] to check whether the signature of a recent bottleneck could be detected in the different subsamples at times T1 and T2. No bottleneck signature could be detected at T0 in that focus [50]. We used the unilateral Wilcoxon test as advised by the authors [11, 47] in the software documentation. As recommended ([14], pp. 104–105), we assumed infinite allele model (IAM), two-phase model (TPM) with default values (*i.e.*, 70% of stepwise mutation model (SMM) and a variance of 30), and SMM models of mutation. We inferred the occurrence of a bottleneck signature if the test was highly significant with IAM, and significant with TPM, at least. Alternatively, a slightly significant bottleneck signature only observed with IAM more probably reflects small effective subpopulations sizes. We used Bottleneck v 1.2.02 [47] to undertake these tests in each cohort separately.

## Results

### Sex-ratio within samples, and between times (T)

There was an overall and highly significant biased sex-ratio in favor of females (Table 1). This sex-ratio significantly varied between control times in Maro (*p*-value = 0.0078), due to its three times increase at T2 (Table 1). The surface area of Maro (*S*_GPS_, as defined in the section “Effective population densities”) was 128 km^2^. Densities were 0.52, 0.63 and 0.32 of captured flies per km^2^ for T0, T1, and T2, respectively. The number of captured flies did not drop significantly (all *p*-values > 0.06), though half as many flies were captured between T1 and T2. No real differences of SR could be seen between T0 and T1 (*p*-value = 0.8725), which displayed a strongly female biased SR (SR ≈ 0.4 for both, *p*-value < 0.0002) (weak or no effect of the control campaign). At T2, the SR became not significantly different from 1 (Table 1). There was a significant effect of control on the SR (*p*-value = 0.0218, and *p*-value = 0.0507, for T0/T2 and T1/T2 SR comparisons, respectively).

### Population genetics of tsetse flies from Maro before control (T0)

According to previous analyses [50], subdivision was very small: *F*_ST-FreeNA_′ = 0.0434 in 95%CI = [0.0069, 0.0716], with Meirmans’ method, corresponding to a global number of effective immigrants *N*_e_*m* = 5.06 on average and over all the focus. Updated population sizes in traps averaged *N*_e_traps_ = 28 in minimax = [17, 46]. This yielded very small effective population densities in the focus (Table 1). Taking the whole focus as a single unit, effective population size was *N*_e-all_ = 28 in minimax = [20, 36].

Isolation by distance signature was weak (slope of the regression *b* = 0.0074 in 95%CI = [−0.0024, 0.0169]) and only significant with the *D*_CSE_ based Mantel test (*p*-value = 0.02). Between traps, the dispersal distance was *δ*_traps_ = 14–21 km per generation, for GPS and GEPL estimates, respectively with a minimax = [7, 27] km, excluding infinity. Here infinity may translate into a free dispersal on the whole surface area of the focus, which was 33 km long.

### Population genetics of tsetse flies from Maro six generations after the commencement of control (T1)

Subdivision analysis found no significant effect of traps (*p*-value = 0.8077) at T1. If not specified otherwise, we then considered the whole focus as a single population.

We found three locus pairs (8%) in significant LD (*p*-values < 0.0129), none of which stayed significant after BY adjustment (*p*-values > 0.1202).

In the whole focus, considered as a single unit, there was a highly significant (*p*-value < 0.0002) heterozygote deficit: *F*_IS_ = 0.124 in 95%CI = [0.042, 0.226]. With traps as subsample units, *F*_IS_ = 0.123 in 95%CI = [0.038, 0.229], which was not significantly smaller (*p*-value = 0.3118). This confirmed the absence of a Wahlund effect when considering all flies of the focus as a single population. The correlation between the number of missing data and *F*_IS_ was substantial but marginally not significant (*ρ* = 0.5193, *p*-value = 0.076), and the corresponding regression explained 45 % of *F*_IS_ variations across loci. No SAD test (undertaken with *F*_IS_) appeared significant (smallest *p*-value = 0.0671), even with the weighted regression (smallest *p*-value = 0.0837). According to Brookfield’s second method, null alleles explained very well all heterozygote deficits. Some loci even displayed more missing genotypes than necessary, *i.e.*, than the expected number of null homozygotes if null alleles explained the heterozygote deficit in a pangamic population. Some loci (Gff3, Gff4, Gff8, Gff16, Gff18, and Gff27) displayed a tendency for stuttering, and significantly so for two of these (Gff16 and Gff18). Even though null alleles explained rather well the heterozygote deficits, stuttering correction (see Appendix B) worked well for all loci but Gff16. Locus Gff16 was in fact perfectly explained by null alleles (13 missing data were expected with Brookfield’s second method, and 12 were observed). The resulting *F*_IS_ = 0.085 in 95%CI = [0.003, 0.206] was still highly significant (*p*-value = 0.0002). We excluded loci that displayed too many missing data and a low *F*_IS_ (Gff4, Gff8, Gff21 and Gff23) (*i.e.*, for which missing genotypes did not correspond to null homozygotes). With the remaining loci, missing data explained almost all *F*_IS_ variations (*ρ* = 0.9487, *p*-value = 0.0257, *R*^2^ = 0.9973). Alternatively, loci without or with very rare null alleles displayed a non-significant *F*_IS_ = −0.001 in 95%CI = [−0.041, 0.027] (*p*-value = 0.9716).

Using data corrected for stuttering and traps, effective population size was *N*_e*-*traps_ = 187 in minimax = [14, 523] across methods. It was infinite for all traps with Estim, but with two lower limits 13 and 19, that we used for the average. Effective population density was *D*_e-traps-GPS_ = 1.46 in minimax = [0.11, 4.09] flies/km^2^, hence not really different but more variable than at T0 (Table 1), and at least not smaller. When we considered Maro as a single population, *N*_e-all_ = 83 in minimax = [17, 181].

For isolation by distance, we recoded missing data as null homozygotes (999999) only for loci significantly affected by null alleles: *i.e.*, Gff3, Gff12, Gff16 and Gff18. With the 95%CI of the slope, isolation by distance between traps was not significant: *b* = 0.012 in 95%CI = [−0.0233, 0.0694], but the Mantel test with *D*_CSE-FreeNA_ was highly significant (*p*-value < 0.0001). Using the whole sample based effective population density found in the focus, we inferred dispersal distances *δ*_traps_ = 4 km in 95%CI = [2, Infinity], where “Infinity” means a free, or almost free, dispersal of flies across the zone. This may correspond to the distance between the two most distant traps with at least one fly, hence *D*_geo-max_ = 39 km.

### Population genetics of tsetse flies from Maro nine generations after control (T2)

Taking traps as subsample units, only a single locus pair displayed a significant LD (*p*-value = 0.0059), which did not stay significant after BY adjustment (*p*-value = 0.8867). There was a significant heterozygote deficit *F*_IS_ = 0.117 in 95%CI = [0.002, 0.246] (*p*-value < 0.0002). The ratio between the standard error of *F*_IS_ and *F*_ST_ was *r*_StdrdErr_ = 4.6, which suggests genotyping errors (null alleles and/or SAD). No SAD signature could be found (smallest *p*-value = 0.2418). A positive correlation was found between *F*_IS_ and *F*_ST_ (*ρ* = 0.2833, *p*-value = 0.2315), and between *F*_IS_ and the number of missing data (*ρ* = 0.5963, *p*-value = 0.0451). Missing data explained 16% of *F*_IS_ variations. There was a non-significant signature of subdivision: *F*_ST_ = 0.022 in 95%CI = [−0.001, 0.049] (*p*-value = 0.1159). Pooling flies of all traps into a single subsample produced a significantly higher *F*_IS_ = 0.138 in 95%CI = [0.02, 0.275] as compared to the one measured within traps (*p*-value = 0.04). This means that pooling traps produced a significant Wahlund effect, although the proportion and significance of LD tests did not increase (one significant *p*-value = 0.022). So, contrarily to T0 and T1 samples, we could not ignore the traps for further analyses. Stuttering detection and cure was only efficient for locus Gff8 (Appendix B) and we thus kept recoding for that locus only. With this new dataset, *F*_IS_ = 0.104 in 95%CI = [−0.008, 0.234] (*p*-value < 0.0002). The correlation between *F*_IS_ and missing data was improved (*ρ* = 0.7826, *p*-value = 0.0063, *R*^2^ = 0.2023). Subdivision was still not significant but marginally so (*F*_ST_ = 0.025 in 95%CI = [0.001, 0.052], *p*-value = 0.0865). Recoding missing data and using FreeNA correction, we obtained *F*_ST-FreeNA_ = 0.0305 in 95%CI = [0.0043, 0.0618]. The correlation between *H*_S_ and *G*_ST_ was strongly negative and significant (*ρ* = −0.8167, *p*-value = 0.0054). We thus used Recodedata to get a maximized subdivision dataset and compute a standardized subdivision index. With FreeNA correction for null alleles, the standardized *F*_ST-FreeNA_′ = 0.1413 in 95%CI = [0.0257, 0.2352]. In an Island model of migration, this would correspond to a number of immigrants of *N*_e_*m* = 1.5 in 95%CI = [0.8, 9.5] individuals per subpopulation (trap) and generation.

Over the 10 traps with at least one genotyped fly, the effective population size averaged *N*_e_traps_ = 18 in minimax = [15, 23] individuals across methods. Given the weakness of subdivision between traps, this corresponded to an estimate of the global effective size of the whole zone at T2. Effective population density of the whole focus was obtained *D*_e__traps-GPS_ = 0.14, in minimax = [0.12, 0.18] individuals/km^2^. Taking the focus as a single population we obtained *N*_e_all_ = 171 in minimax = [15, 394]. Effective population size estimate from traps provided no value with sibship frequencies, only one value for LD and Estim, three for coancestries and seven with the heterozygote excess. The average was thus fairly biased toward the last method. Estimate based on the whole focus provided values for all methods for all times and minimax values for almost all of those but coancestries (no estimate for T2) and LD for which maximum values were all infinite. Following this, it is probable that *N*_e_All_ is more reliable than *N*_e_traps_.

Isolation by distance between traps was not significant with the 95%CI of the slope (*b* = 0.003 in 95%CI = [−0.0013, 0.0093]), or with the *D*_CSE_ based Mantel test (negative slope, *p*-value = 0.4951). Thus at T2, subdivision, if any, became disconnected from geography and we could consider a free, or almost free dispersal in the whole focus.

### Genetic differentiation between trapping times

The results of this analysis are presented in Table 2. There was no significant signature of genetic differentiation between trapping times. For these analyses, we kept the initial coding of alleles (no correction for stuttering), but we still used correction for null alleles using FreeNA, as described in the Material and Methods section.

**Table 2:** Results of the *G*-based test of differentiation between different trapping times for *Glossina fuscipes fuscipes* from Maro, combined across all traps for each time pair (*p*-value) and adjusted with BY FDR correction (*p*-BY). Corresponding average *F*_ST_ corrected for null alleles are also given with their 95% confidence intervals between brackets.

Time pairs	*p*-value	*p*-BY	*F*_STFreeNA_
T0/T1	0.6874	1	0.0526 [−0.0050, 0.1090]
T0/T2	0.2092	1	0.0415 [−0.0198, 0.1116]
T1/T2	0.6562	1	0.0264 [−0.0239, 0.0861]

### Population genetics at different times considering Maro as a single demographic unit

**Figure 2:**
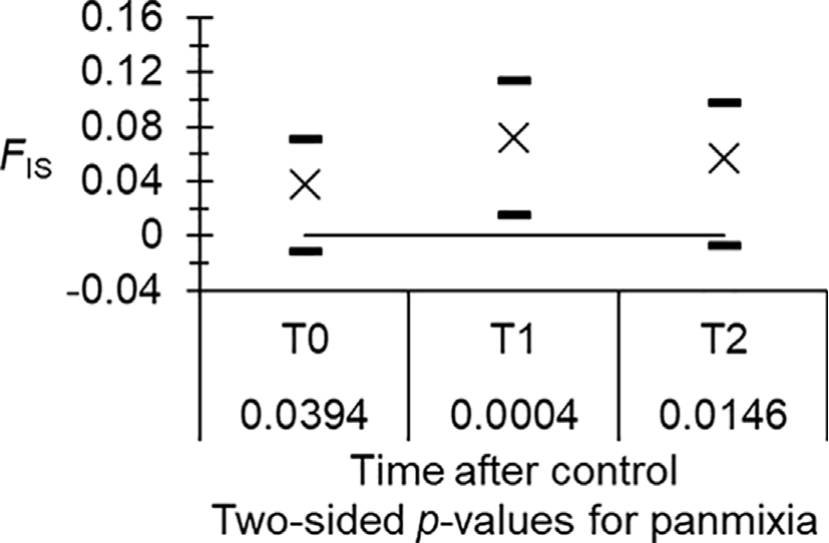
Heterozygote deficits (*F*_IS_, crosses) of *Glossina fuscipes fuscipes* before (T0) and after (T1 and T2) the beginning of control, and considering the focus of Maro as a single demographic unit. Black dashes are the 95% confidence intervals computed with 5,000 bootstraps over loci, and results of testing for panmixia are below time labels. Here, data were corrected for stuttering at loci Gff16 and Gff18.

Analyzing the three subsamples (T0, T1 and T2), we confirmed the role of stuttering for loci Gff16 and 18. After stuttering correction (for Gff16: alleles 144 and 166, and 156 to 166 were pooled; for Gff18: alleles 212 and 214 and 220–228 were pooled), null alleles explained most, if not all, *F*_IS_ observed at the different loci. There were 1, 4 and 2 pairs in significant LD in T0, T1 and T2, respectively. At T0, all BY corrected probabilities *p*_BY_ = 1; at T1 all *p*_BY_ > 0.1623; and at T2, only one locus pair (GFF12–GFF23) stayed significant after BY correction (*p*_BY_ = 0.0451). The heterozygote deficit increased between T0 and T1 or T2 (Figure 2), but never significantly so (*p*-values = 0.125 and 0.248, respectively).

**Figure 3:**
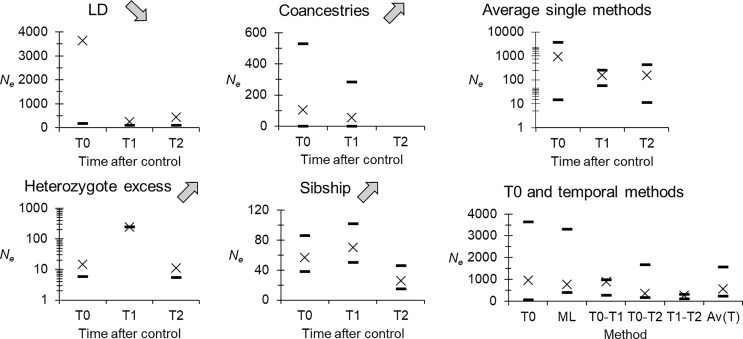
Effective population size (*N*_e_) in *Glossina fuscipes fuscipes* from the human African trypanosomosis focus of Maro in southern Chad before (T0) and after the beginning of control (T1 and T2), for different methods (crosses). These figures were all computed considering the whole focus as a single population (noted *N*_e_All_ in the text). Confidence intervals (dashes), as described in the material and methods section, are 95% confidence intervals, except for average over single methods where dashes correspond to averaged minimum and maximum values. Grey arrows indicate the evolution of *N*_e_ after control (increase or decrease) expected in case of a Wahlund effect due to the recolonization by foreign flies, and according to the method used. Absence of crosses or dashes means “infinite”. Here, data were corrected for stuttering at loci Gff16 and Gff18.

Effective population sizes are presented in Figure 3. At T0, the average was *N*_e-All_ = 955 in minimax = [15, 3643]. Except for Coancestries and T2, evolution of *N*_e_ was in the direction expected for samples experiencing a Walhund effect signature, if some flies recolonized the focus from remote sites with different allele frequencies at T1. Nevertheless, the grand average seemed unchanged, and temporal methods provided a result that was consistent with single sample methods (Figure 3).

**Figure 4:**
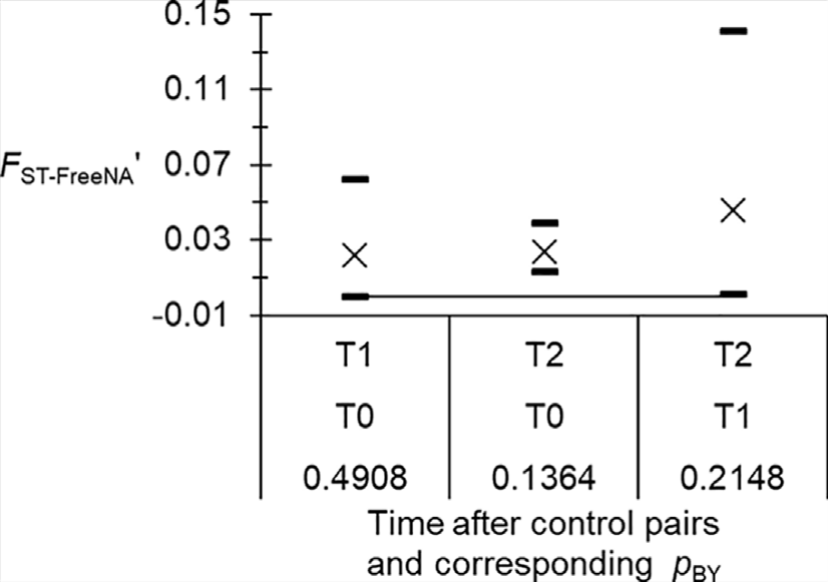
Measure of genetic differentiation, corrected for null alleles and excess of polymorphism (*F*_ST-FreeNA_′), between pairs of time of sampling before (T0) and after (T1 and T2) for *Glossina fuscipes fuscipes* from the Maro focus (Chad), and randomization test results corrected with the BY procedure (*p*_BY_). Here, data were corrected for stuttering at loci Gff16 and Gff18.

As can be seen in Figure 4, genetic differentiation, even when the 95%CI of *F*_ST-FreeNA_′ was above 0, was never significant at the BY level between any paired times before and/or after the beginning of control. So, at best, genetic differentiation between T0, T1 and T2 was weak.

### Factorial components analysis and DAPC analysis at the scale of southern Chad

**Figure 5:**
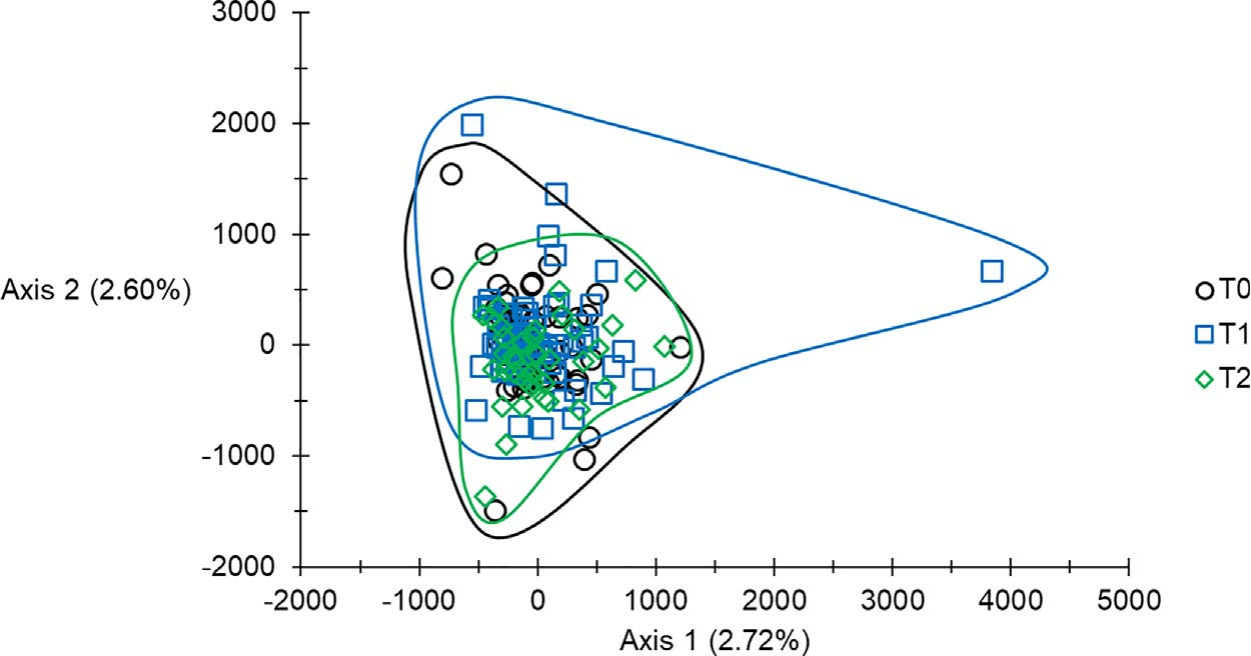
Presentation of the two dimensions projection of individuals of *Glossina fuscipes fuscipes* from Maro (southern Chad), sampled at different times before and after the beginning of control (T0, T1 and T2), and according to the first two axes of a factorial correspondence analysis. Percent of inertia are indicated. No axis was significant according to the broken stick. Here, data were corrected for stuttering at loci Gff16 and Gff18.

The results of the FCA analysis are presented in Figure 5. No axis was significant according to the broken stick. The cloud defined by flies from T1 was the most heterogeneous, followed by T0, and then by T2. Several outliers suggested recruitment of flies from remote sites, either from remote locations of unknown origins or nearby sites. Nevertheless, most flies captured at T1 and T2 presented a “local” genetic profile.

### Bottleneck detection

No bottleneck signature was found in Maro at T0, unlike the other zones investigated [50]. This investigation was undertaken without stuttering correction and considering each trap as a subpopulation. According to the results presented in Table 3, stuttering correction, or increased subsample sizes, allowed us to remove this inconsistency, as Maro displayed a significant bottleneck signature at all times, even if less significantly so at T1. Undertaking the same test with the uncorrected data with all loci, or removing the two loci with significant stuttering, in fact provided results that were very similar, or even more significant ones. Sample sizes were probably why Maro did not display a significant bottleneck signature in [50].

**Table 3:** Result of the bottleneck signature detection in *Glossina fuscipes fuscipes* from Maro before control (T0) and after the beginning of control (T1 and T2) for different models of mutation (IAM: infinite allele model; TPM: two-phase model; SMM: stepwise mutation model), as indicated by the different *p*-values.

Time after control	IAM	TPM	SMM
T0	0.0020	0.0098	0.5449
T1	0.0029	0.0645	0.5898
T2	0.0020	0.0244	0.7871

## Discussion

The main aim of the present study was to assess the impact of vector control, through tiny target deployment, on the population biology of tsetse flies, using population genetics tools. This work is part of a broader program on vector control actions against tsetse flies in different countries affected by *Glossina* borne diseases of humans and animals [3, 42] with similar approaches. In Chad, among the two documented HAT foci (Mandoul and Maro) [42], the consequences of tiny target deployment on the population biology of treated populations could be assessed only in Maro where enough flies could be trapped after control had begun (T1 and T2 of the present study), while only two or no flies could be captured in Mandoul after tiny targets deployment [38].

The deployment of sentinel traps did not vary across dates. Consequently, trapping performances at T0, T1 and T2 can be compared, even though these possibly do not fully reflect the real demographic state of the population of tsetse flies in Maro. The density of trapped flies increased slightly between T0 and T1, but dropped to half the initial value at T2. Strong female biased SRs affected the densest samples at T0 and T1, but the SR was not significantly different from T1 at T2, which is in variance with the positive effect of density on SR that was suggested in another study in Chad [50]. This suggests that at T2, more males than females survived control in this focus and/or immigrated from outside to fill the spots emptied by vector control. In the absence of any further information, any interpretation about the SR of captured flies would be speculative. From a previous study in different zones of southern Chad [50], the SR measured in traps probably reflects environmental driven dispersal differences between males and females that translate into differences in probability to be caught. The real deterministic causes of SR will need to be explored by further and specific research. Regarding the density of captured flies and SR, control had no effect at T1, and displayed a much more appreciable one at T2. Since traps capture hunting flies, and females feed more than males, as they need to produce L3 larvae [50], vector control measures are expected to affect females more than males. Hence, an increase in SR should be observed after an efficient vector control campaign. This may explain the significant increase observed at T2.

The genetic composition of flies captured in Maro one year and one and a half years after the beginning of the control campaign was difficult to interpret. Most flies were probably local survivors. Nevertheless, several parameter variations observed during the different analyses at T1 and T2 suggest a complex pattern of recolonization. Differences in isolation by distance or subdivision measures, effective population densities, absence of significant genetic differentiation between temporally spaced samples after several generations (3 generations between T1 and T2, 6 between T0 and T1, and 9 between T0 and T2), similarities in heterozygosity, effective population sizes, and bottleneck signatures all suggested limited impact of the control campaign on the tsetse population structure. Nevertheless, non-significant but convergent independent signatures of weak Wahlund effects also suggested partial recolonization by flies from more remote zones, with different alleles frequencies. This was also suggested by the FCA analysis. With the relatively large effective population sizes we observed, recolonization by a majority of flies from the local population, as suggested by our results, is in line with the weak differentiation observed between dates of sampling, even after nine generations. Sample sizes at T1 and T2 represented all flies that could be capture at these dates. Nevertheless, with nine highly polymorphic loci and relatively large sample sizes, even modest genetic differentiation would have been detected here, as was the case for spatial differentiation with smaller sample sizes [50]. Moreover, an impact of vector control on the population genetics of tsetse flies could be detected, with the same number of loci and similar subsample sizes (at least after control had begun), but with deployment of tiny targets on the whole area, in *G. palpalis palpalis* Robineau-Desvoidy, 1830 in the focus of Bonon in Côte d’Ivoire. Alternatively, in the focus of Boffa (Guinea), where tiny targets cannot be deployed everywhere in the Mangrove against *G. palpalis gambiensis* Vanderplank, 1949, with eight loci and similar subsample sizes, and despite a significant reduction in apparent densities per trap, no effect could be observed on the population biology of the targeted tsetse population, and using the same kind of data analyses (revised preprint re-submitted to *PCI Infections* (https://www.biorxiv.org/content/10.1101/2023.07.25.550445v2)).

The south border of Chad with the CAR, which was not investigated on the CAR side, represents many potential unexplored, and possibly tsetse rich environments and thus potential sources for reinvasion with tsetse flies. The Maro focus will thus need special attention, with particular care regarding its most southern parts. Given the high potential for dispersal of *G. f. fuscipes* in these environments, this may also represent an encouragement to sustain surveillance, and to expand it to CAR, should this be possible. This is in variance with the results obtained with the vector control campaign in the more isolated Mandoul focus, where no tsetse could be captured after 2015, one year after the beginning of control [38].

In conclusion, this analysis confirmed that the Maro focus appears to be at high risk of reinvasion. Successful and sustainable interruption of transmission of g-HAT will thus require continuous control and surveillance, particularly regarding the southern part of the country, at the CAR border, where the epidemiological status of g-HAT is unknown and where the unstable political situation obscures the future of disease control in this particular geographic area. Importantly, tiny target strategies, even if deployed only in accessible spots, have proven to be very efficient at protecting humans against tsetse bites and trypanosome infections [6, 12]. For now, continuous similar control measures are advised to protect people from g-HAT in the Chadian part of the Maro focus. The nagana status of Maro has not yet been explored, but one can safely assume that such measures are also protective of animals and should thus also benefit the local economy.

## Supplementary material

The supplementary material of this article is available at https://www.parasite-journal.org/10.1051/parasite/2024013/olm. *Supplementary File S1*: Raw data.

## Data Availability

Examples of scripts to compute geographic distances and surface areas with the package geosphere are available in Appendix A.

## Appendix A

### Example of scripts to compute geographic distances or surface areas with the R package geosphere

# to compute geographic distance (in meters) with GPPS coordinate in decimal #degrees

distGeo(c(long1, lat1),c(long2, lat2))

#with two files with two columns (longitude and latitude), the first file #containing the GPS coordinates of the first point of site pairs, and the second #file containing the corresponding GPS coordinates of the second point of site #pairs.

LongLat1 <- read.table("Long1Lat1.txt", header=TRUE, stringsAsFactors=TRUE, sep="\t", na.strings="NA", dec=".", strip.white=TRUE)

LongLat2 <- read.table("Long2Lat2.txt", header=TRUE, stringsAsFactors=TRUE, sep="\t", na.strings="NA", dec=".", strip.white=TRUE)

distGeo(LongLat1, LongLat2)

# To compute the area of a polygon in angular coordinates (longitude/latitude) #on an ellipsoid.

#Dataset has two columns: Longitude and Latitude

Dataset <- read.table("MyData.txt", header=TRUE, stringsAsFactors=TRUE, sep="\t", na.strings="NA", dec=".", strip.white=TRUE)

attach(Dataset)

areaPolygon(data.frame(Longitude,Latitude))

## Appendix B

### Strategy used to detect and correct for the presence of stuttering

#### T1 subsample

We chose to try correcting stuttering for all loci with a heterozygote deficit for one repeats size difference to check if it improved the *F*_IS_. For locus Gff3, we pooled alleles 198–202 with 196; for Gff4, we pooled alleles 142–152 with 140; for Gff8, 156–162 with 156, and 176–190 with 174; for Gff16, 158–170 with 156; for Gff18, 214 with 212, and 222–228 with 220; and for Gff27, 169–171 with 167, 187–199 with 185, and 205–207 with 203.

We then recomputed all *F*_IS_’s, to check for an improvement of the data after such stuttering corrections. For Gff3, *F*_IS_ = 0.092 after correction against 0.142 before; for Gff4, *F*_IS_ = −0.008 after correction against *F*_IS_ = 0.009 before; for Gff8, *F*_IS_ = −0.087 after correction against 0.029 before; for Gff16, *F*_IS_ = 0.558 after correction against 0.441 before; for Gff18, *F*_IS_ = 0.162 after correction against 0.348 before; and for Gff27, *F*_IS_ = 0.047 after correction against 0.148 before. Except for locus Gff16, all loci were thus improved after stuttering correction.

#### T2 subsample

There was a weak signature of stuttering for loci Gff4, Gff16 and Gff18 and a stronger one (one significant test) for Gff8. We recoded alleles at these loci in the following way: for Gff4, we pooled alleles 146–152 with allele 144, 158–160 with 156, and 170 with 168; for Gff8, 158 with 156, 162 with 160, 176 with 174, 180 with 178, and 184 with 182; for Gff16, 158 with 156, 162 with 160, and 166 with 164; and for Gff18, 214 with 212, 220–222 with 218, and 226–230 with 224. Analysis of this modified dataset revealed that only recoding Gff8 provided a beneficial result and we kept the recoding for this locus only.
